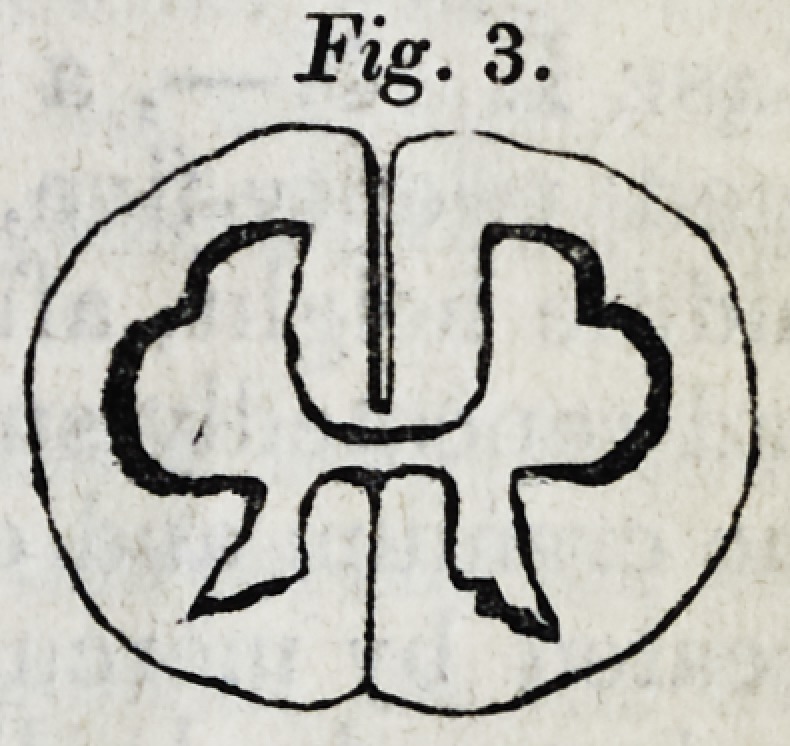# Some Remarks upon the Distribution of the Grey Matter in the Spinal Chord, and upon the Relative Proportions of the Anterior and Posterior Segments of the Chord at Different Parts

**Published:** 1832-06

**Authors:** Herbert Mayo

**Affiliations:** Professor of Anatomy in King's College, &c.


### ANATOMY OF THE SPINAL CHORD.

*Some Remarks upon the Distribution of the Grey Matter in the Spinal Chord, and upon the relative Proportions of the anterior and posterior Segments of the Chord at different Parts.* By HERBERT MAYO, F.R.S., Professor of Anatomy in King's College, &c.

THE following measurements were made upon the spinal chord of a remarkably muscular body; the age about twenty-five years, the height five feet four inches.

The entire length of the spinal chord, from the commencement of the decussation of the anterior pyramids to its lumbar end, was 18.5 inches. The fine terminal production of nervous matter at the inferior end of the chord extended .62 of an inch below the origin of the last nerve.

The length of the medulla oblongata, from the beginning of the decussation to the inferior border of the pons Varolii, was 1.26 inches.

The breadth of the spinal chord at the origin of the second cervical nerve was .5 of an inch; its depth, .4.

Breadth at the interval between the fifth and sixth cervical nerves, .57; depth, .4; depth of the anterior median furrow, .15. (Vide Fig. 1.)

Breadth at the thinnest part of the chord in the back, (viz. four and a half inches above the origin of the last nervous filament,) .36; depth, .34; depth of the anterior median furrow, .14. (Vide Fig. 2.)

Breadth where the chord is thickest towards its inferior termination, (viz. at two inches above the origin of the last nervous filament,) .46; depth, .38; depth of the anterior median furrow, .18. (Vide Fig. 3.)

The three figures which follow are representations, twice the size of nature, of the appearances seen upon making sections of the spinal chords at the three last points adverted to in the preceding measurements. The upper part of each figure is supposed to be the anterior surface of the spinal marrow.

*Fig.* 1.

*Fig.* 2.

*Fig.* 3.

It is shewn in these figures,

1. That the grey matter in either half of the spinal cord, is an irregular capsule of cineritious matter, containing white matter, (resembling therefore the corpus fimbriatum in either olivary body and arbor vitæ), not, as it is commonly supposed to be, a *solid* pillar of cineritious matter.

2. That the form of the capsule of grey matter differs remarkably at different parts of the chord.

3. That, at the lower part of the chord, from which the nerves arise which are distributed to the lower extremities, (in which strength of muscular action is the characteristic endowment,) the anterior production of the grey matter is principally developed. (Vide Fig. 3.)

4. That the greater proportional and actual depth of the anterior median furrow at the lower part of the spinal chord marks, no less than the disposition of the grey matter, the superior development of the anterior or *motor part* of the spinal chord in this region.

5. That, at the upper part of the chord, where the nerves arise which are distributed to the upper extremities, (in which the muscular force is less, but the fineness of sensation perhaps greater than in the lower,) the anterior part of the grey matter is less than at the ~~upper~~ part of the chord, while the posterior production is proportionately greater. (Vide Fig. 1.)

The disposition of the grey matter in the medulla oblongata is already accurately figured in my plates of the brain: I have therefore not given a representation of sections of that part in the present notice. In the kangaroo, and in the horse, I find the figure of the grey matter in the different parts of the spinal chord greatly to resemble that in man.

*King's College; May* 15*th*, 1832.

## Figures and Tables

**Fig. 1. f1:**
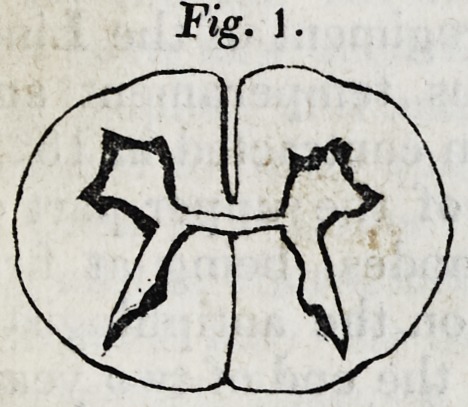


**Fig. 2. f2:**
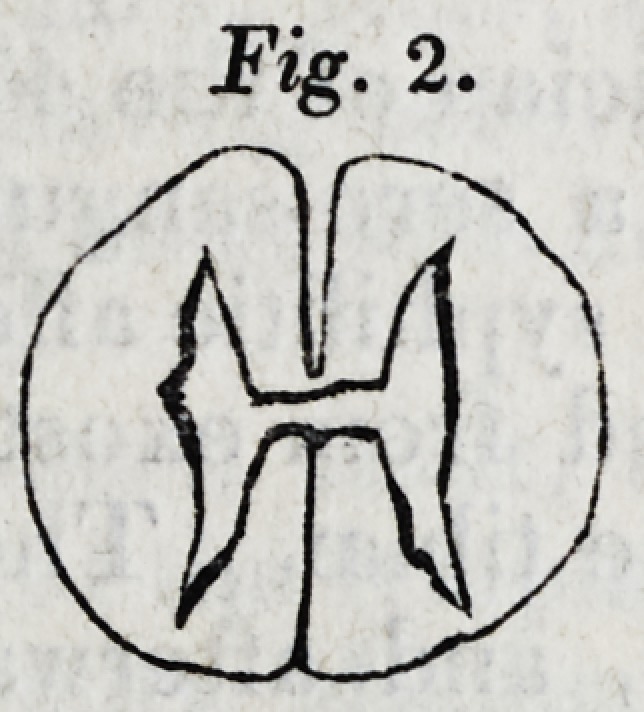


**Fig. 3. f3:**