# Erratum

**Published:** 1832-07

**Authors:** 


					in the last Number.?P. 468, tenth line fr' a top, for ** upper, read (t lower."

				

